# Upregulation of *Integrin-α6* and *Integrin-β1* Gene Expressions in Mouse Spermatogonial Stem Cells after Continues and Pulsed Low Intensity Ultrasound Stimulation

**DOI:** 10.22074/cellj.2018.4286

**Published:** 2017-11-04

**Authors:** Mahdi Mohaqiq, Mansoureh Movahedin, Manijhe Mokhtari Dizaji, Zohreh Mazaheri

**Affiliations:** 1 Department of Anatomical Sciences, Faculty of Medical Sciences, Tarbiat Modares University, Tehran, Iran; 2 Department of Medical Physics, Faculty of Medical Sciences, Tarbiat Modares University, Tehran, Iran

**Keywords:** Colonization, Proliferation, Stem Cell, Ultrasound

## Abstract

**Objective:**

low intensity ultrasound (continues and pulsed) is a form of energy. Spermatogonial stem cells (SSCs) are at the base of male fertility. This study investigated the effects of low intensity ultrasound stimulation (LIUS) and low intensity pulsed ultrasound stimulation (LIUPS) on the expression of germ cell-specific and pluripotency genes in SSCs *in vitro*.

**Materials and Methods:**

In this experimental study, isolated SSCs from neonatal male mice were cultured in Dulbecco’s Modified Eagle’s Medium (DMEM) with 10% fetal bovine serum (FBS). In addition, to confirm identification of SSCs, PLZF protein was detected positively in SSCs derived colonies. SSCs were stimulated by LIUS and LIUPS for 5 days, followed by assessment of expression of *integrin-α6 (Itga6)* and *β1 (Itgβ1)*, as two germ cell-specific genes, and Oct- 4, as a pluripotency gene, on day 21st by quantitive reverse transcriptase-polymerase chain reaction (qRT-PCR). To investigate the proliferation rate and colonization of SSCs in different groups, counting whole number of the cells and colonies as well as analysis of the respective diameters were performed on days 7^th^, 14^th^ and 21^st^. Data was analyzed by ANOVA test.

**Results:**

LIUS and LIUPS treatment of mouse SSCs increased expression of *Itga6* and *Itgβ1* genes in the experimental groups, compared to the control group (P<0.05), whereas there was no significant difference between the groups, regarding the expression of *Oct-4* gene. These treatments maintained survival rate, while they increased proliferation rate and colonization of SSCs during the first week of culture. However, within the second week, proliferation rate and colonization were decreased in the experimental groups.

**Conclusion:**

These results suggested that LIUS and LIUPS treatment had good effect on SSCs proliferation and colonization, based on the gene-specific marker expression during 21 days culture *in vitro*.

## Introduction

Spermatogonial stem cells (SSCs) are at the base of spermatogenesis. Evidences show reduction of SSCs in some cases of infertility. Enrichment and proliferation of SSCs *in vivo* and *in vitro*, is important and colonization of spermatogonial cells is a crucial step to treat infertility, germ cell gene change, cell transfect and differentiation of SSCs in vitro. SSCs content is similar to the others cells, including basement membrane, cytoplasm, nucleus and etc. The plasma membrane of SSCs has very important function in receiving signals, induction and transportation of modified signals into cytoplasm or nucleus. Integrins are heterodimeric transmembrain proteins, consisting of α and β subunits which provide a link between the extracellular matrix (ECM) and the intracellular cytoskeletal components as well as actin filaments. Integrins are thought to function through undergoing conformational changes, activating them and revealing their ligand binding site. Mechanical stress activates this pathway, thus the cells can react to the changes in their physical environment (1). Ultrasound waves produce pressure and transmit to adherent cells through interactions with the ECM. Ultrasound is widely used as a diagnostic imaging tool, while low intensity ultrasound (LIUS) and low intensity pulsed ultrasound (LIUPS) promotes bone and tissue repair processes by stimulating cells growth and inducing proliferation and differentiation of some cells (2). The precise mechanism by which LIUS and LIUPS mediates such effect is not clearly defined. In previous studies LIUS and LIUPS have been reported to cause increased proliferation rate on human umbilical cord-derived mesenchymal stem cells (2), hematopoietic stem cells (3), adipose-derived stem cells (4) and SSCs (5, 6) in 7 days culture. These waves also increased the expression of *Cbfa-1/Runx2, Igf-receptor, Alk-3, Alkaline phosphatase, Osteopontin, TGF-β1 *and *BMP-7* in rat bone marrow stromal cells (7). They upregulated expression of some genes like *c-Jun, c-Myc, Cox-2, Egr-1, Tsc-22* as well as *Osteonectin and Osteopontin* in rat osteoblastic cells (8). It has been determined that these waves could elevate the expression of CAT and PAL genes in hazal cells (9). However, the effect of LIUS and LIUPS on expression of germ cellspecific and pluripotency genes of SSCs, which have very important function in male fertility, has not yet been explored. SSCs are at the foundation of spermatogenesis and male fertility. SSCs are very rare, representing only 0.03% of all germ cells in rodent testes (10). This is due to differentiation of divided SSCs to spermatogonia, spermatocytes, spermatids and spermatozoa. Culture, enrichment and colonization of SSCs provide a practical approach to investigate testicular transplantation, cell transfect and differentiation of SSCs in vitro, as critical factors for treatment of infertility (11). In this study we investigated the effect of LIUS and LIUPS on the expression of *integrin alpha 6 (Itga6)* and *beta 1 (Itgβ1)* genes as well as colonization, proliferation and survival rate of SSCs during 21 days of culture.

## Materials and Methods

### Isolation and culture of spermatogonial stem cells

In this experimental study, spermatogonial cells were obtained from neonate male mice NMRI. All stages of this research are based on the approval of Ethics Committee of Tarbiat Modares University (Tehran, Iran). They were maintained under standard conditions with free access to food and water. In order to obtain the SSCs, we used a modified method published by Javanmardi et al. (12). Briefly, a decapsulated testis was cut into small pieces and seminiferous tubules were transferred to dulbecco’s minimum essential medium (DMEM, Gibco, UK) containing collagenase IV (0.5 mg/ml, Sigma, USA) and incubated in 37˚C for 20 minutes, then centrifuged 5 minutes with 1500 rpm speed. The medium on top of palette was subsequently exchanged with phosphate buffered saline (PBS, Gibco, USA) and then centrifuged twice for 3 minutes in 1000 rpm. This phase caused to delete interstitial tissue from testis pieces. Trypsin (0.5 mg/ml, Sigma, USA) was next added to this solution for 2 minutes and then centrifuged for 5 minutes in 1500 rpm. Eventually the obtained cells were pooled. Obtained mixture, commonly included two kinds of cell: spermatogonial cells and sertoli cells.

### Identification of spermatogonial stem cells using immunocytochemistry

PLZF protein (marker for SSCs) was detected in the SSCs derived colonies by immunocytochemistry, 7 days after culturing. The procedure of immunocytochemistry was performed according to previous study (13). Briefly, the cells were grown on the glass slides and fixed for 20 minutes in 4% paraformaldehyde at room temperature, before rinsing with PBS. After permeabilization by 0.2% Triton X-100 (MP Biomedicals, USA) for 1 hour to facilitate antibody penetration, the slides were washed with PBS supplemented with 0.2% bovine serum albumin. Nonspecific antigens were blocked with 10% normal goat serum (Vector Laboratories, USA). The slides were then incubated overnight at 37˚C with a mouse monoclonal anti-PLZF antibody (diluted 1:100; Santa Cruz Biotechnology, USA). The slides were washed with PBS and then the second antibody (goat Texas redconjugated anti-mouse IgM, diluted 1:100; Sigma, USA) was applied for 2 hours at room temperature in the dark.

### Design of study

#### Ultrasound device preparation

**Fig.1:**
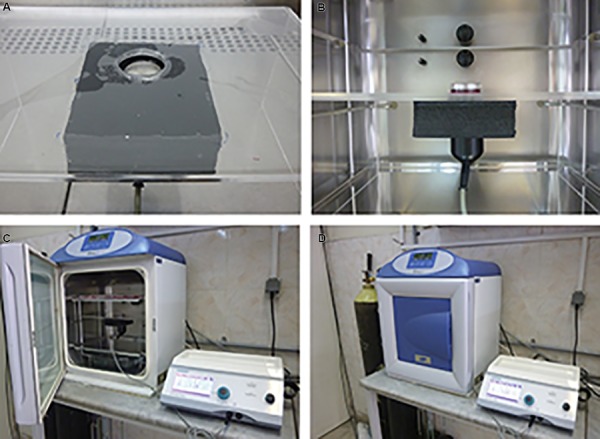
Experimental set-up of the ultrasound-mediated spermatogonial stem cells (SSCs) stimulation. A, B. Position of ultrasound transducer and cell culture plate, C. and D. Orientation of ultrasound stimulation set-up in incubator.

Our ultrasound device (Physiomed, Germany) was adjusted with the following parameters: ultrasound frequency: 1 MHz, intensity: 200 mW/cm^2^, time: 200 seconds, duration: 5 days. These design parameters were chosen based on the previous studies (3, 5, 6). Ultrasound stimulation was applied by a transducer to spermatogonial cells cultured in an enclosed sterile conventional 3.5 cm cell culture plate, incubating at 32˚C temperature and 5% CO_2_ (Fig .1). It was transmitted through the bottom of the well via coupling gel between the transducer and the plate. Based on the previous study (5), during SSC stimulations, no more than 1˚C temperature change was observed.

#### Spermatogonial stem cells stimulation by ultrasound

Cells were maintained in DMEM supplemented with 10% (v/v) fetal bovine serum (FBS, Gibco, UK). The cells were exposed to LIUS (1 MHz, 200 mW/cm^2^) and LIUPS (1 MHz, 200 mW/cm^2^ and 40% DC) as experimental groups. The control group was cultured in DMEM containing 10% FBS. After different stimulation modes, SSCs were cultured for 21 days and they were then evaluated. To investigate proliferation rate, the mean number of whole cells per volume was determined every seven days. Obtained colonies from spermatogonial cells were assessed every seven days with invert- phase microscope (Zeiss, Germany), equipped by ocular grid.

#### Quantitative reverse transcriptase polymerase chain reaction

*Oct-4* expression was evaluated as the pluripotency gene in SSCs and the spermatogonial markers of *Itga6* and *Itgβ1* were evaluated by quantitative reverse transcriptase polymerase chain reaction (qRT-PCR). Total RNA was extracted from spermatogonial cells in different groups on days 0 and 21, by RNX-PlusTM (Cinnagen, Iran) according to the manufacturer’s recommendations. In order to remove genomic contamination, RNA was treated with DNase I Fermentase kit (Lithuania) based on the protocol described by manufacturer. Concentration of total RNA was determined using UV spectrophotometer (DPI-l, Qiagen, IRI). cDNA was synthesized from 1000 ng RNA sample with a Revert AidTM first-strand cDNA synthesis kit (Fermentase, Lithuania) using oligo (dT) primers. qRT-PCRs were carried out using Master Mix (Cinnagen, Iran) and CYBER Green I (Fluka, Switzerland) in an Applied Biosystems StepOneTM instrument (Applied Biosystems, USA). PCR program was started with an initial melting cycle, 4 minutes at 94˚C, to activate the polymerase and followed by 40 cycles as follow: a melting step (20 seconds at 94˚C), an annealing step (30 seconds at 57˚C), and an extension step (20 seconds at 72˚C). After completing the PCR run, quality of the reactions was confirmed by melting curve analyses. For each sample, the reference gene (*Gapdh*) and the target gene were amplified in the same run. The comparative CT method (2^-ΔΔCT^) was used to determine the relative quantification of target genes and normalized to a housekeeping gene (*Gapdh*) and related to a calibrator (0 day of SSCs). A validation of experiment was performed to verify that the target efficiency and reference was approximately equal.

### Statistical analysis

One-way ANOVA and Tukey post tests were used to determine the statistical significance of determined differences in the mean values among experimental groups, using the SPSS statistical software (SPSS 16.0 production mode facility). The data are presented as mean ± SD. Each data point represents the average of three separate experiments with three repeats in each experiment. P<0.05 indicated statistical significance.

### Ethical consideration

Current study was conducted under the protocol approved by the animal experimentation committee of Medical Sciences Faculty in Tarbiat Modares University.

## Results

### Spermatogonial stem cells isolation and culture

**Fig.2:**
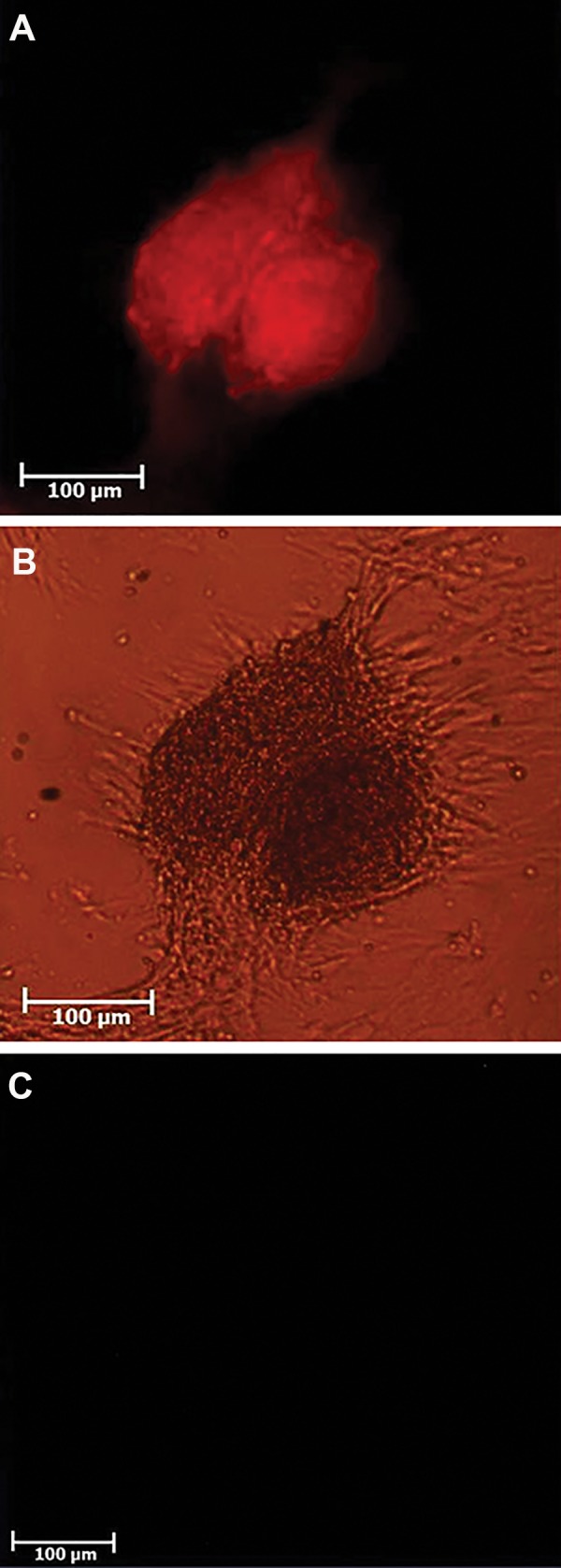
Detection of PLZF positive cells, using immunoflurecent staining, in spermatogonial stem cells (SSCs) derived colonies. A. Red florescent cells are PLZF positive in the obtained colonies, observing under immunofluorescent microscope, B. Colonies observed under inverted phase-contrast microscope, and C. Negative control group. These cells were observed under immunofluorescence microscope.

Isolated cells (spermatogonial and sertoli cells) were cultured in DMEM supplemented with 10% FBS. On the first day of culture, SSCs were single. Following two days, the majority of round cells was adherence and aggregated in to clumps, and then colonies were formed on day 5. These cell colonies were formed completely and their size were increased. In addition, PLZF protein was detected in the obtained colonies from these cells (Fig .2).

### Evaluation of proliferation rate and colonization after ultrasound stimulation

**Fig.3:**
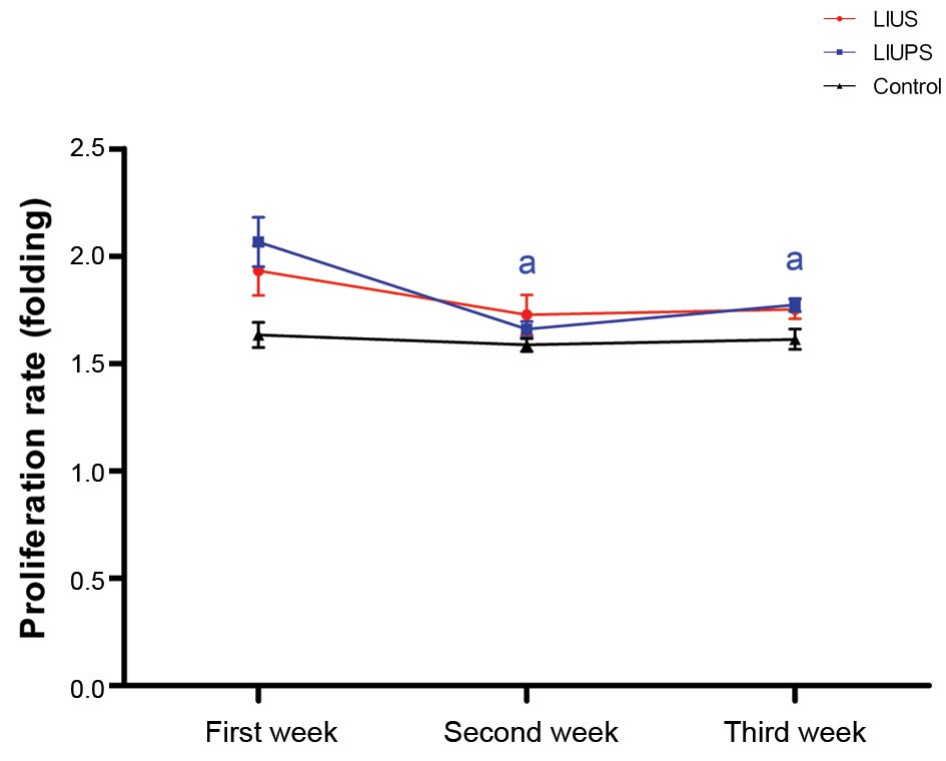
Proliferation rate in the experimental groups during three weeks culture. a; Refer to significant differences compared to the other weeks in the same group (P<0.05).

**Fig.4:**
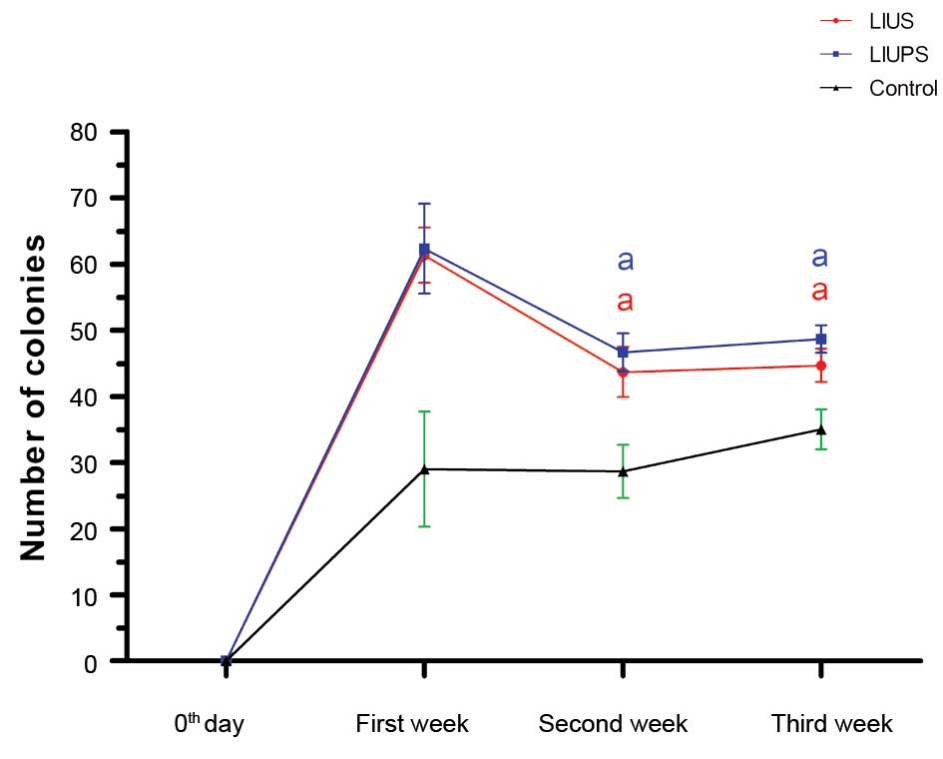
The number of spermatogonial stem cells (SSCs) colonies in the experimental groups during three weeks culture. a; Refer to significant differences compared to the other weeks in the same group (P<0.05).

**Fig.5:**
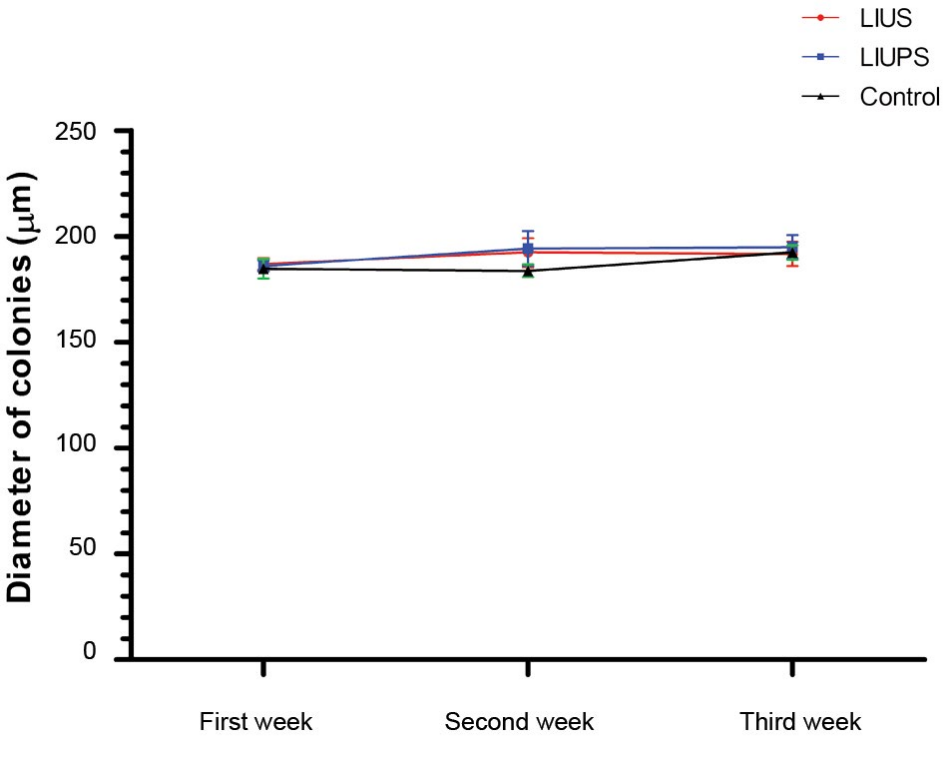
Diameter of spermatogonial stem cells (SSCs) colonies in the experimental groups within different times.

We evaluated the influence of different modes of low intensity ultrasound stimulation on proliferation rate and colonization in SSCs compared to the control group. The results showed that all of the two modes of low intensity ultrasound stimulation, significantly increased (P<0.05) proliferation rate in SSCs within the first week. However, proliferation rate was decreased significantly (P<0.05) within the next week, in LIUPS stimulated group, compared to the first week (Fig .3). Our results also showed that LIUS and LIUPS stimulation increased the number of SSCs colonies during 21 days of culture, although this trend was not continued and the colony quantity was significantly decreased in the next week (P<0.05) (Fig .4). Furthermore, our results indicated no significant difference at the size of colony diameters in different experimental groups and times (Fig .5).

### Quantitative reverse transcriptase polymerase chain reaction

**Fig.6:**
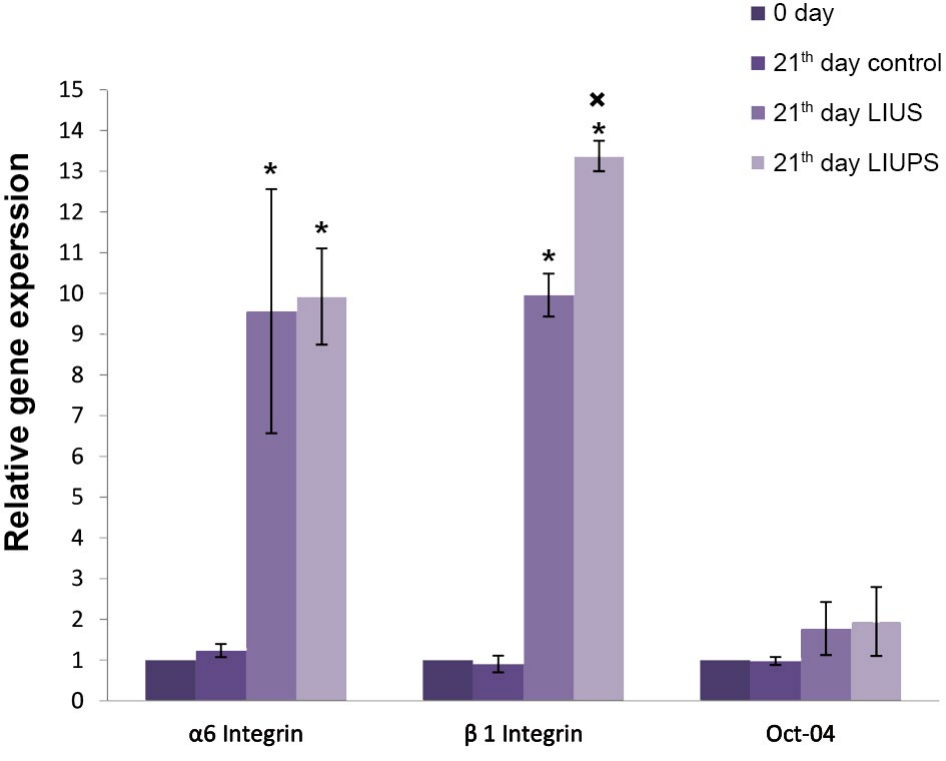
The ratio of germ cell-specific genes and pluripotency gene expression. Relative expression was shown as the ratio of target gene to *Gapdh*. *; Significant difference between different times, in the same gene (P<0.05) and ×; Significant difference of LIUS group stimulation on day 21st, compared to the other groups in same gene (P<0.05).

qRT-PCR was performed in different groups of the isolated SSCs to analyze the expression of a pluripotency marker subset, as well as germ cell-specific genes. Results demonstrated that the expressions of *Itga6* and *Itgβ1* were significantly increased (P<0.05) in LIUS and LIUPS groups in comparison with control group on the 21st day and the beginning of culture. In addition, the level of the *Itgβ1* expression was increased (P<0.05) in LIUPS group in comparison with LIUS group on the 21^st^ day. No significant difference of *Oct-4* gene expression was determined between experimental and control groups (Fig .6).

## Discussion

In this study, we investigated the effects of LIUS and LIUPS on germ cell specific and pluripotency genes as well as colonization, proliferation and survival rates of SSCs during 21 days of culture *in vitro*. We applied LIUS and LIUPS stimulation to culture SSCs in DMEM supplemented with 10% FBS with 200 mW/cm^2^ intensity for 200 seconds during 5 days. On day 21^th^, we found that expressions of *Itga6* and *Itgβ1* were increased in the experimental groups. However, stimulation with LIUPS mode resulted in a better expression of Itgβ1 in SSCs compared to LIUS mode and unstimulated SSCs. Consistent with present data, an earlier study demonstrated that stimulating osteoblasts and chondrocytes with low intensity of ultrasound transiently increased the expression of specific integrins, namely α5 and β1 (14). At the first week of culture, we found that proliferation rate and number of colonies were increased in the experimental groups. However, this increase was not continued and we observed that proliferation and colonization rates were decreased on the following weeks. Our findings obtained from in vitro study strongly revealed that LIUS and LIUPS stimulation could improve the number of mouse SSCs and their colonies. Some reports indicated that LIUPS stimulates proliferation rate and colonization in hematopoietic stem/progenitor cells (HSPCs) (3). Xu et al. (3) stimulated hematopoietic stem precursor cells with low intensity pulsed ultrasound for 4 days and reported that it can enhance proliferation rate and burst forming unit-erythroid colony formation on day 5. Korstjens et al. (15) suggested that the LIUPS stimulates chondrocyte proliferation and matrix production in human articular cartilage *in vitro* (15).

Mohaqiq et al. (5, 6) stimulated mouse SSCs by LIUS and LIUPS and reported that the proliferation and colonization rates were increased during 7 days of culture *in vitro*. Integrins provide a link between ECM and intracellular cytoskeletal components as well as actin filaments. Integrin proteins are thought to have function by undergoing conformational changes that activate them and reveal their ligand binding site. Integrins can bind to cytoskeletal components and other signaling molecules, while they activate several intracellular signaling pathways in response to mechanical stress such as sound waves, thus enabling the cells to react to changes in their physical environment (1). Integrins protein family acts as sensitive mechanoreceptors on the surface of cells. Ultrasound waves produce mechanical stimulation which has been transferred to adherent cells via interactions with the ECM. Increase in integrin expression was observed in the cells after LIUPS treatment. It was shown to activate a number of downstream kinases including focal adhesion kinase (FAK), phosphatidylinositol 3-kinases (PI3K) and mitogens activate protein kinase (16), indicating that LIUS with their effect on transmembrain proteins, such as integrins, might be able to stimulate more SSCs to divide and it can enter these cells to mitotic process through regulation of self-renewal or differentiation pathway. Our results of LIUS and LIUPS effects on SSCs showed that these waves had a useful shorttime effect on proliferation and colonization during 21 days culture. We did not observe any increase on the proliferation and colonization rates, compared to the first week of culture. Unfortunately, previous studies of LIUS or LIUPS effect on cells proliferation and colonization did not report data from culture, more than one week. In addition, we investigated LIUS and LIUPS effect on SSCs survival rate.

## Conclusion

We have demonstrated a novel LIUS and LIUPSmediated effect on SSCs proliferation, colonization and survival rates during 21 days culture. We also concluded that LIUS and LIUPS stimulation increased *Itga6* and *Itgβ1* expressions, as two genes playing critical role in SSCs proliferation and differentiation. Hence, LIUS and LIUPS stimulation could be a good strategy for improving the efficiency and fate of stem cell transplantation, gene therapies, in addition to improve efficiency and outcome of stem cell enrichment.
